# Spam1-associated transmission ratio distortion in mice: Elucidating the mechanism

**DOI:** 10.1186/1477-7827-3-32

**Published:** 2005-08-10

**Authors:** Patricia A Martin-DeLeon, Hong Zhang, Carlos R Morales, Yutong Zhao, Michelle Rulon, Barry L Barnoski, Hong Chen, Deni S Galileo

**Affiliations:** 1 Department of Biological Sciences, University of Delaware, Newark, DE 19716, USA; 2 Department of Anatomy and Cell Biology, McGill University, Montreal, Quebec, Canada; 3 Department of Genetics, Thomas Jefferson University, Philadelphia, PA, USA

## Abstract

**Background:**

While transmission ratio distortion, TRD, (a deviation from Mendelian ratio) is extensive in humans and well-documented in mice, the underlying mechanisms are unknown. Our earlier studies on carriers of spontaneous mutations of mouse Sperm Adhesion Molecule 1 (Spam1) suggested that TRD results from biochemically different sperm, due to a lack of transcript sharing through the intercellular cytoplasmic bridges of spermatids. These bridges usually allow transcript sharing among genetically different spermatids which develop into biochemically and functionally equivalent sperm.

**Objectives:**

The goals of the study were to provide support for the lack of sharing (LOS) hypothesis, using transgene and null carriers of Spam1, and to determine the mechanism of Spam1-associated TRD.

**Methods:**

Carriers of Spam1-Hyal5 BAC transgenes were mated with wild-type female mice and the progeny analyzed for TRD by PCR genotyping. Sperm from transgene and Spam1 null carriers were analyzed using flow cytometry and immunocytochemistry to detect quantities of Spam1 and/or Hyal5. Transgene-bearing sperm with Spam1 overexpression were detected by fluorescence in situ hybridization. In wild-type animals, EM studies of in situ transcript hybridization of testis sections and Northern analysis of biochemically fractionated testicular RNA were performed to localize Spam1 transcript. Finally, AU-rich motifs identified in the 3' UTR of Spam1 RNA were assayed by UV cross-linking to determine their ability to interact with testicular RNA binding proteins.

**Results:**

The Tg8 line of transgene carriers had a significant (P < 0.001) TRD, due to reduced fertilizing ability of transgene-bearing sperm. These sperm retained large cytoplasmic droplets engorged with overexpressed Spam1 or Hyal5 protein. Caudal sperm from transgene carriers and caput sperm of null carriers showed a bimodal distribution of Spam1, indicating that the sperm in a male were biochemically different with respect to Spam1 quantities. Spam1 RNA was absent from the bridges, associated exclusively with the ER, and was shown to be anchored to the cytoskeleton. This compartmentalization of the transcript, mediated by cytoskeletal binding, occurs via protein interactions with 3' UTR AU-rich sequences that are likely involved in its stabilization.

**Conclusion:**

We provide strong support for the LOS hypothesis, and have elucidated the mechanism of Spam1-associated TRD.

## Introduction

A remarkable feature of mammalian testicular maturation of sperm is the syncytial organization that results from the presence of intercellular cytoplasmic bridges among the germ cells. These bridges allow transcript sharing among genetically different spermatids and provide a mechanism by which these cells develop synchronously into biochemically and functionally equivalent sperm [1]. Studies of spermatid-expressed genes for Protamine [2] and several X-linked sperm-specific proteins [3] provide strong evidence for transcript sharing. However sharing may not be a global phenomenon for all spermatid-expressed genes, particularly those encoding membrane proteins [4]. Moreover there is compelling evidence for functionally different sperm in a male leading to TRD, as best exemplified by mice carrying different alleles at the *t*-complex [5,6]. The TRD seen for the *t*-haplotypes has been explained by unequal sharing of post-meiotic products [1], but there is no evidence for this mechanism.

Earlier our laboratory provided evidence for a Lack-of-Sharing hypothesis (LOS) for TRDs that were discovered in the progeny of Robertsonian (Rb) translocation-bearing mice [7-9], and shown to be associated with carriers of spontaneous mutations of the murine *Sperm adhesion molecule1 (Spam1) *gene [10,11]. *SPAM1 *encodes a widely conserved sperm membrane protein [12] which has multiple essential roles in mammalian fertilization [13]. The murine gene which maps to proximal chromosome 6 [14] in a cluster of hyaluronidase genes containing *Hyalp1*, *Hyal4*, and *Hyal5 *[15], is spermatid-expressed and the RNA is transcriptionally regulated since it first appears together with the protein in the testis of postnatal Day 21.5 mice [10]. The TRDs were seen in heterozygotes of either of two Rb translocations, Rb(6.15) and Rb(6.16), in which multiple *Spam1 *point mutations were shown to be present [11], leading to reduced expression of both the RNA and the protein [10]. We have since observed that in these mice *Hyalp1 *and *Hyal5 *which have overlapping functions with *Spam1 *also have point mutations that would have contributed to the TRDs [16]. Furthermore, the fact that *Spam1 *null mice are fertile suggests that other hyaluronidases are able to compensate for this gene [17].

Our LOS hypothesis for the *Spam1*-related TRDs is based on our finding of compartmentalization of the RNA, as assessed by RNA-FISH [10]. This compartmentalization precludes transcript sharing in normal as well as mutant mice, and leads to biochemically different sperm with respect to the protein. Importantly, the protein is inserted in the acrosomal membrane soon after formation [10]. Our study showed that in males carrying different alleles of *Spam1*, compartmentalization leads to biochemically and functionally different sperm and the resulting TRD [10]. The objectives of this study were to use transgene and null carriers of *Spam1 *to garner support for the LOS hypothesis and to study the transcript localization in normal mice to gain insights into the underlying mechanism leading to the TRD.

## Materials and methods

### Breeding and Transmission Study

The studies were approved by the Animal Care Committee at the University of Delaware and conform to the guide for the Care and Use of Laboratory animals published by the National Institutes of Health (publication 85-23, revised 1985). A 150 kb mouse BAC (bacterial artificial chromosome) clone, described earlier [14] and after sequencing shown to contain *Spam1 *and *Hyal5 *and their regulatory regions, was used to generate transgenic mice, as reported elsewhere [Zhang et al., submitted]. Male trangenic founders, Tg8, Tg9, and Tg11, and their F_1 _progeny were mated to C57BL females, and transgenic and non-transgenic offspring identified by PCR genotyping at weaning. Tail DNA samples from progeny were screened using BAC vector-specific primers (F-5'AACATACGAGCCGGAAGCAT 3' and R-5'GATTCAGGTTCATCATGCCG 3') for PCR. Additionally, four females which were mated with Tg8 carriers or wild-type C57BL males (the background for the transgenic mice) were examined for resorption sites on the 14^th ^day of pregnancy.

Hemizygotes or carriers for *Spam1 *null were generated by mating C57BL males with *Spam1 *null females. Null mice were obtained from the laboratory of Tadashi Baba where they were generated [17]. Backcross matings were then set up by pairing sexually mature hemizygous null males with *Spam1 *null females.

### Flow Cytometry and Immunoflourescence

#### Flow Cytometric Analysis

Flow cytometry was performed to quantify the amount of Spam1 on the sperm surface. Caput and/or caudal sperm from adult wild-type C57BL mice and carriers of Tg8, Tg11, and the *null *allele were collected in PBS and fixed in 1.5% paraformaldehyde for 1 hr at RT. After washing and blocking in 2% BSA in PBS they were stained using the rabbit antipeptide mouse Spam1 antiserum generated from a C-terminal 15-mer (#381 – 395) oligopeptide (custom made by Zymed, San Francisco, CA) (diluted 1:400) specific for Spam1 [18,19]. The secondary antibody was FITC-conjugated goat anti-rabbit IgG (diluted 1:320). After several washes of the cells, fluorescence was measured (up to 30,000 sperm for each sample) using a FACScan (Becton-Dickinson, San Jose, CA) flow cytometer with a Lysis II software package. The FACScan instrument uses an argon laser at 488 nm with detectors for FITC. Prior to preparing for cytometric analysis, an aliquot of the sperm suspension was used for indirect immunofluorescence of Spam1.

#### Indirect Immunofluorescence

Caput and caudal sperm recovered from adult wild-type C57BL mice, and Tg8 and Tg11 hemizygotes and homozygotes were fixed and processed as above. They were then stained with rabbit antipeptide mouse Spam1 or Hyal5 antiserum generated from a 20-mer at C-terminal #474–492 (custom made by Zymed, San Francisco, CA) (diluted 1:400). Peptide blocking of the antiserum showed that the signal was specific. The secondary antibody was FITC-conjugated goat anti-rabbit IgG (diluted 1:320). Controls were incubated with preimmune rabbit/FITC-conjugated secondary antibody. The sperm were then mounted on slides in ρ-phenylenediamine antifade with 1.5 μg/ml of 4' 6-diamidino-2-phenylindole (DAPI) for standard fluorescence microscopy. The specimens were examined using a Zeiss Axiophot (Carl Zeiss, Oberkochen, Germany) with the appropriate FITC filter set, and imaged using a CCD-cooled camera and IPLab software. A total of 200 sperm were examined from each group to identify abnormal expression of Spam1. The analysis of Hyal5 was qualitative only.

### Flow sorting of Sperm and Fluorescence *In Situ *Hybridization

#### Sorting

After flow cytometric analysis, sperm were sorted in preparation for fluorescence *in situ *hybridization. A sorting gate was set on the histogram to collect sperm from the half of the bimodal distribution with the higher fluorescence intensity. Sperm were sorted on the FACSCalibur in exclusion mode at an approximate sort rate of 200 events/second and collected into 50 ml tubes in PBS. They were then pelleted and fixed with methanol-acetic acid.

#### Fluorescence *In Situ *Hybridization

BAC DNA was labeled by nick-translation using SpectrumRed™ direct-labeled dUTP (Vysis, Downers Grove, IL). Methanol-acetic acid fixed sperm were treated with 10 mM dithiothreitol in 0.1 M Tris-HCl for 45 min on ice to decondense the chromatin. They were then dipped in ddH_2_O before air-drying and hybridization was as described [14].

### *In Situ *Transcript Hybridization

CD-1 mice (n = 3 per group) were anesthetized with sodium pentobarbital, and the testes were perfused through the left ventricle with 4% paraformaldehyde, 0.1 % glutaraldehyde, and 3% dextran sulfate in 0.05 M phosphate buffer (pH 7.4) for 15 min. Following perfusion, the testes were removed and immersed in the same fixative for 5 hr at 4°C. The tissues were then cut into small blocks of approximately 8 mm^3^, embedded in 2.5% melted agarose (at 60°C), and cut into 60 μm-thick frontal sections with a vibrotome. Groups of 10 sections were collected in autoclaved vials and washed three times in RNAse-free 0.05 M phosphate buffer (pH 7.4) at room temperature. Glycine (1 M) was added to the buffer to neutralize aldehyde groups.

Prehybridization and hybridization procedures were performed as described previously [20]. Briefly, testicular sections were transferred from the phosphate buffer to the prehybridization buffer containing 4 × SSC (1 × SSC is 0.15 M NaCl plus 0.015 M sodium citrate) and 1 × Denhardt's solution for 1 hr at room temperature with gentle agitation. Sections were then immersed in hybridization buffer containing 1 ml of 8 × SSC, 1 ml of deionized formamide, 100 μl of Sarkosyl (2.3 mg/ml), 200 μl of 1.2 M phosphate, and 1.50 μg per vial of ^3^H-labeled *Spam-1 *antisense probe (specific activity 1.47 × 10^7 ^cpm/μg) or 1.50 μg per vial of a ^3^H-labeled control sense probe (specific activity 1.57 × 10^7 ^cpm/μg). The antisense probe was generated from a unique PCR fragment from the 3' UTR of *Spam1 *and thus would not cross-hybridize with other hyaluronidases. After hybridization overnight at 40°C, the sections were rinsed sequentially at the same temperature in 4 × SSC and 0.1 × SSC for 1.5 hr. Following the washes, the sections were quickly dehydrated in 50%, 70%, 90%, and 100% ethanol and embedded in Epon.

### Radioautography

Ultrathin sections (65 nm thick) were cut from selected areas of seminiferous tubules within the epon blocks for electron microscope radioautography. The sections were placed on celloidin coated glass slides, coated and dipped in Ilford L4 emulsion according to the method of Kopriwa [21]. After 3 months exposure, the sections were developed in a solution physical development, which produce round silver grains [22]. The sections were then transferred to electron microscopy nickel grids, immersed for 45 sec in glacial acetic acid to remove the celloidin and carbon films.

#### Quantitative Analysis

For selected steps of spermiogenesis 10 EM micrographs, corresponding to 10 different cells per testis/animal, were selected for analysis according to the method of Nadler [23]. In most of the cases 50 silver grains were scored over each step spermatid cytoplasm. Since 85% of the silver grains were associated to the ER, a circle with a radius of 20 mm (equivalent to 0.23 μm resolution at 60,000x) was centered over silver grains that did not overlay any organelle. When an organelle was found within the circle, the radioautographic silver grain was attributed to such an organelle and considered as "exclusive". If the circle included more than one organelle the silver grains was considered "not exclusive". According to Haddad et al. [24] and Nadler [23] this procedure permits identification of the source of radioactivity, with a probability of 95%. For quantitative analysis steps spermatids were grouped as follow: steps 1–5 and 6–8 (round spermatids), steps 9–11 (early elongated spermatids), steps 12–16 (late elongated spermatids).

### Biochemical fractionation of *Spam1 *RNA

Testicular RNA was extracted from sexually mature CD-1 males and free cytosolic-, cytoskeleton-bound, and membrane-bound fractions were separated by subcellular fractionation techniques as described [25]. Northern blotting was then performed with the fractions and hybridization carried out sequentially using *Spam1 *and β-*actin *^32^P-labeled probes.

### RNA Probe labeling and *in vitro *label transfer Assay by UV cross-linking

A 77 bp 3' UTR fragment (nts 1909–1985) of *Spam1 *cDNA containing AU-rich elements (AREs) was obtained by PCR and cloned into pSTBlue-1 vector according to the manufacturer's instructions (Novagen, Madison, WI). Sense RNA probe was generated by T7 RNA polymerase transcription of the *Hind*III-linearized plasmid in the presence of digoxigenin-11-UTP (DIG) using an *in vitro *transcription system [Riboprobe^® ^System (Promega, Madison, WI)] in accordance with the manufacturer's protocol.

AU-rich sequence binding protein (AUBP) assays were performed using UV cross-linking (UVXL) label transfer. Testes of wild-type C57BL 4–5 month-old mice were used for cytoplasmic protein extraction as described [26]. To test for specificity of binding, unlabeled antisense DNA oligos (100-fold molar excess) were used in competition assays. The oligos were mixed with the labeled probe at 70°C for 10 min and renatured for 1 hr at 22°C before adding the protein extract. The label transfer was performed as described [25,26] with slight modifications. Briefly, 40 μg of cytoplasmic protein extract was incubated with 5 ng of digoxigenin-labeled RNA for 30 min at 22°C in a reaction volume of 20 μl with cytoplasmic extraction buffer. Subsequently, RNase T1 (0.3 U) was added to the mixture for 10 min at 22°C, followed by heparin (final 5 μg/μl) for 10 min at 22°C. The mixture was transferred to a microplate and UV-cross-linked in a GS Gene Linker™ UV Chamber (3 × 10^5 ^μJ, 254-nm bulbs) (BIO-RAD, Hercules, CA) by placing it 1.0 cm from the source for 15 min on ice. The mixture was then incubated with RNase A (final concentration 100 μg/ml) at 37°C for 15 min. Sodium dodecyl sulfate (SDS) sample loading dye was added, samples were boiled for 3 min, subjected to 12.5% SDS-PAGE and then transferred to a nitrocellulose membrane according to standard protocols. Proteins on blots were visualized using the WesternBreeze Chemiluminescent Immunodetection kit (Invitrogen, Carlsbad, CA), following the manufacturer's protocol.

## Results

**Figure 1:**
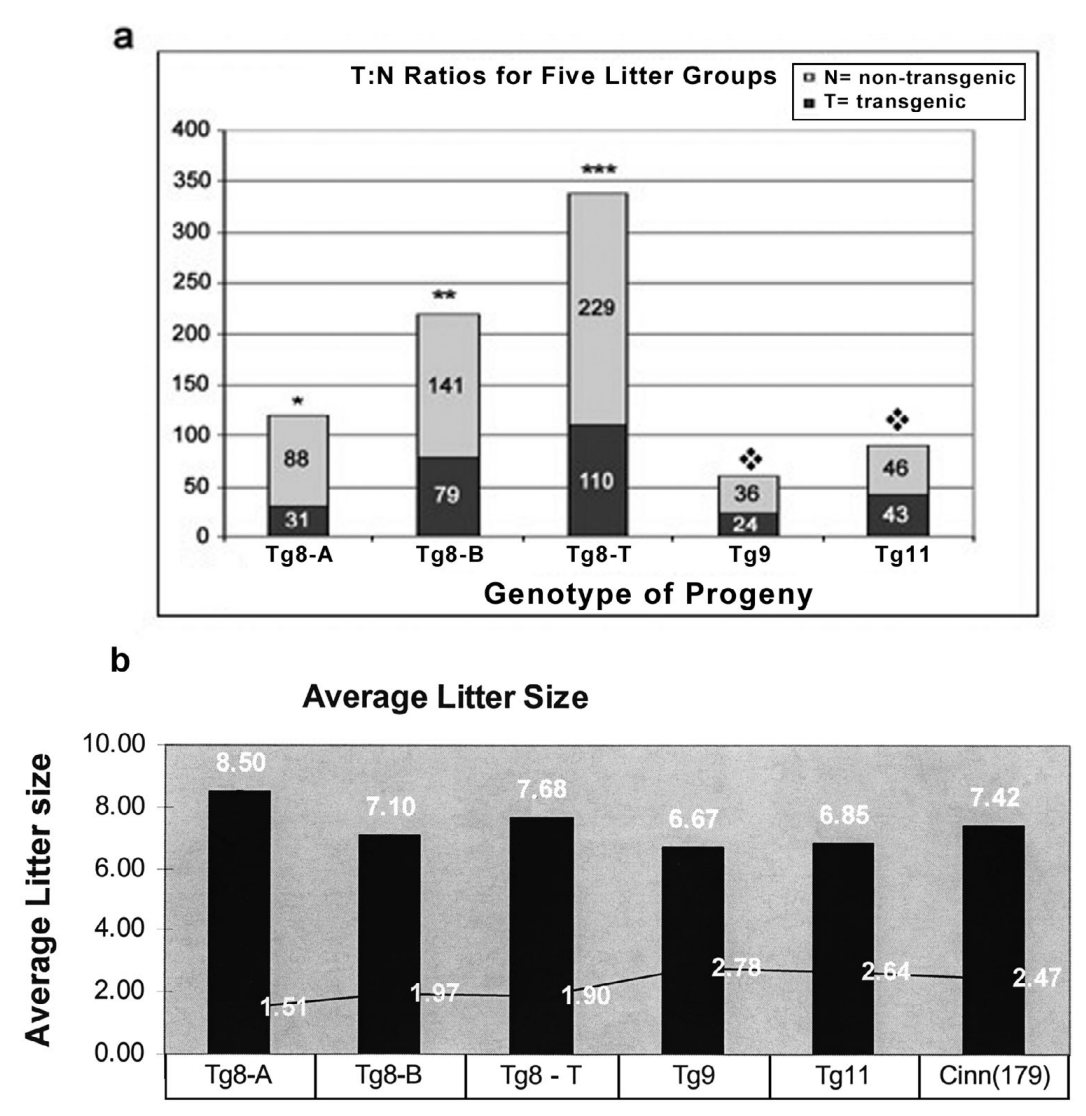
Transmission frequencies of *Spam1-Hyal5 *BAC transgenes in hemizygotes of three lines, Tg8, Tg9, and Tg11, reveal a distortion only for Tg8/+. **a) **Histograms showing the rate of production of transgenic and non-transgenic progeny analyzed at weaning on Day 21. Tg8-A represents the progeny of the founder and 3 F_1 _hemizygous males, while progeny from F_2_-F_4 _hemizygous males are seen in Tg8-B. Tg8-T represents the total progeny analyzed for the Tg8 line. There is a significant deviation from 1:1 for Tg8-A (χ^2 ^= 27.30; P < 0.001), Tg8-B (χ^2 ^= 17.47; P < 0.001), and Tg8-T (χ^2 ^= 41.8; P < 0.001) all represented by an asterisk; while there was no difference in 1:1 ratios (P > 0.05) for Tg9 and Tg11, represented by the diamond. **b) **The TRD for Tg8/+ mice does not result from post-zygotic selection against transgene-bearing zygotes, as revealed by the average litter sizes of transgenic lines. In addition to the BAC transgenic lines we included a *Spam1 *cDNA transgenic line, Cinn (179), which like Tg9 and Tg11 also had a 1:1 ratio in the progeny. The means for the litters ranged from 6.68 to 8.50 and are shown at the top of the histograms with their SDs at the sides. The highest mean, 8.50 ± 1.51, was seen for Tg8A which had the highest TRD, 2.8:1.

We analyzed the progeny of several *Spam1-Hyal5 *BAC transgene carriers (Tg9, Tg11, and Tg8 with transgene-copy numbers of 2, 8, and 10; respectively) for the rate of transmission of the transgenes. While the transgenes were transmitted in Mendelian proportions for Tg9 and Tg11 males (P > 0.05), Tg8 demonstrated a highly significant (P < 0.001) TRD arising from a deficiency of transgene-bearing progeny analyzed at postnatal Day 21 (Fig. 1a). The most severe TRD, 2.8:1 was seen for the progeny of the founder and three transgene-bearing F_1 _males (Tg8A), while males from subsequent generations showed a ratio of 1.8:1 (Tg8-B). The combined population of 339 progeny (Tg8-T) had an overall ratio of 2:1 (P < 0.001) (Fig. 1a), and reveals that the TRD is heritable.

There was no evidence that post-zygotic selection could explain the TRD in Tg8 mice, as the average litter size in Tg8A progeny which had the most severe TRD was the highest, 8.50, among the transgenic lines (Fig. 1b). Further, examination of 14-day fetuses retrieved from matings of four Tg8 hemizygous males with wild-type females showed 0/40 resorptions compared to 3/36 from congenic wild-type males. This indicated that progeny of Tg8 carriers had no greater tendency for post-implantation loss than those for wild-type males. Thus the progeny of Tg8 hemizygotes have a TRD that is likely not due to *in utero *selection, but rather to meiotic drive.

**Figure 2:**
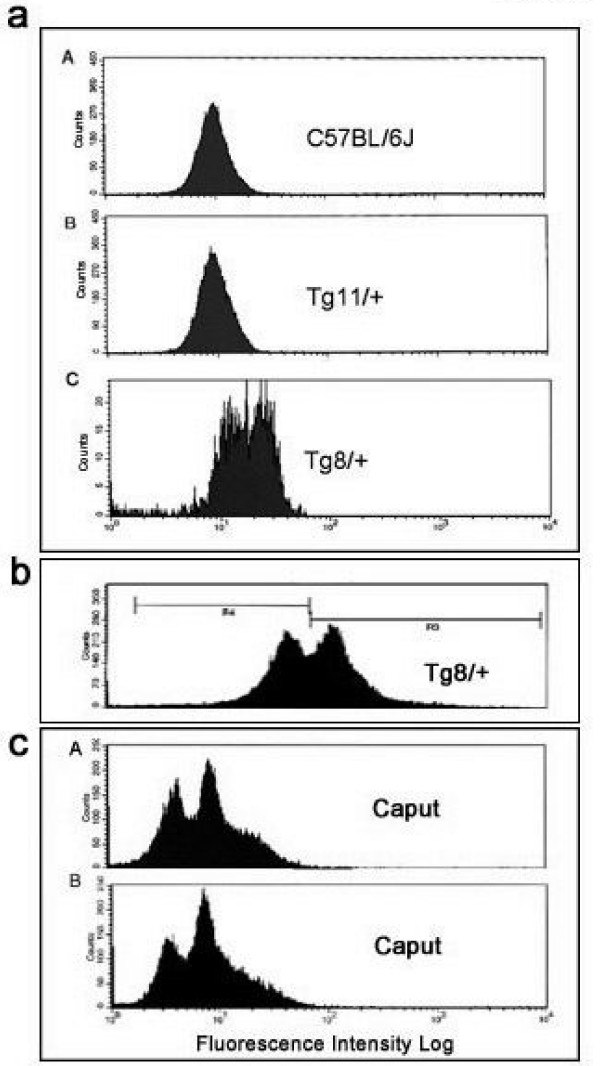
Flow cytometric analyses of sperm from hemizygotes for an overexpressed or a *Spam1 *null allele show bimodal distributions. **a) **Caudal sperm from the congenic wild-type, Tg11/+ and Tg8/+ show a bimodal distribution only for Tg8/+. The second peak (on the right) with the greater intensity in this bimodal distribution indicates the presence of a subpopulation of sperm with overexpression. The distributions for the wild-type and Tg11/+ are unimodal with lower mean intensities, indicating a lack of Spam1 overexpression. **b) **Caudal sperm from Tg8 carriers showing the analysis and gating of the sperm for sorting. **c) **Caput sperm from hemizygous null mice show a bimodal distribution in sperm in A and B. The first peak (on the left) in each shows background levels of fluorescence, likely representing sperm with the null allele.

**Figure 3:**
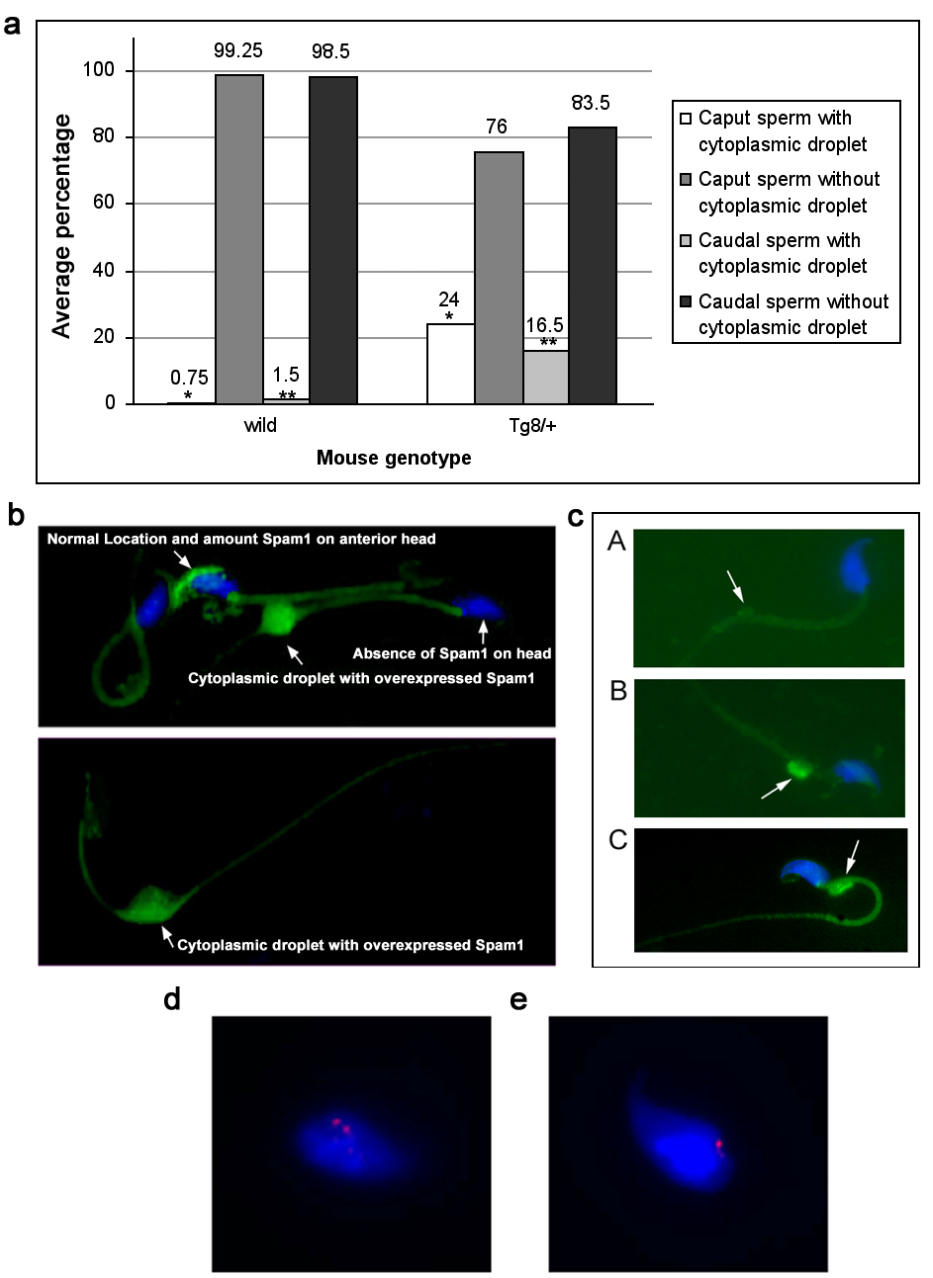
Immunocytochemistry demonstrates that Tg8/+ males have significantly increased numbers of sperm retaining enlarged cytoplasmic droplets (CDs) with overexpressed Spam1 or Hyal5, and a concomitant absence of the proteins on the heads. **a) **Histograms showing the proportion of sperm, from populations of 200, with CDs in wild-type and Tg8/+ males. The greater than 10-fold increase in caudal sperm compared to wild-type is highly significant (χ^2 ^= 10.8; P < 0.01), as is the >30-fold increase in caput sperm (χ^2 ^= 28.5; P < 0.001). **b) **Retention of CDs with overexpressed Spam1 (green staining) in sperm taken from an aliquot used for flow cytometry in Fig. 2a. A sperm with the normal amount and normal location of Spam1 is shown for comparison with the overexpressed protein in the CD. Note that there is non-specific background staining on the tails. **c) **Sperm with overexpressed Hyal5 (green staining) in enlarged CDs near the neck and the absence of the protein on the heads are seen in B and C, while A shows a normal CD without Hyal5. FISH signals on flow sorted sperm showing double signals in transgenic cells **d) **and a single signal in wild-type cells **e)**.

To determine if the sperm population in the Tg8 hemizygotes was heterogeneous with respect to Spam1 expression, caudal sperm from mature males were subjected to flow cytometric analysis. Unimodal distributions of sperm, with similar peaks, were seen for the congenic C57BL/6J wild-type and for Tg11 transgene carriers (Fig. 2a) which showed no TRD. On the other hand, Tg8 carriers showed a bimodal distribution with a shift to the right, indicating that there was a subpopulation of sperm with Spam1 overexpression (Fig. 2a). To corroborate the flow cytometric finding of two phenotypic classes of sperm in Tg8 carriers we performed immunocytochemistry on aliquots of caudal (mature) sperm analyzed using flow cytometry in Fig. 2a. Surprisingly, a highly significant (P < 0.01) proportion of the caudal sperm, (16.5%, Fig. 3a) showed retention of enlarged cytoplasmic droplets (CDs) which were immunopositive for large deposits of Spam1 (Fig. 3b). Sperm with Spam1-containing CDs were lacking the protein on the heads, the normal location, and had far more Spam1 than was found on the heads of normal sperm (Fig. 3b). Thus sperm with the retention of the CDs are consistent with transgenic overexpression of Spam1, as represented by the subpopulation in Fig. 2a with a shift to the right.

We thus studied mice that were homozygous for the Tg8 transgene and observed that CDs were present on 25–30% of caudal sperm, suggesting that in hemizygotes they are associated with the transgene-bearing sperm. To confirm the significant increase of phenotypically abnormal caudal sperm with CDs in Tg8 carriers, we examined immature sperm from the caput of these mice. As might be expected, there were higher numbers of CDs containing large deposits of Spam1 (Fig. 3a) and the difference between the Tg8 and wild-type mice was highly significant (P < 0.001) (Fig. 3a). It should be noted that CDs in wild-type sperm were not only rare, but they were not enlarged (Fig. 3c-A).

To unequivocally demonstrate that the Tg8/+ sperm with large amounts of Spam1 were a result of transgenic overexpression, sperm were sorted after flow cytometric analysis and the subpopulation with the more intense fluorescence (Fig. 2B) recovered for analysis by fluorescence *in situ *hybridization (FISH). The *Spam1-Hyal5 *BAC used to generate the transgenic lines was used as the FISH probe, and 137/217 or 63.1% of the sperm had double hybridization signals representing the transgene and the endogenous locus (Fig. 3d) while 80/217 or 36.9% had a single signal (Fig. 3e). Since double signal due to chromosome 6 disomy has a spontaneous frequency of only 0.9% (our unpublished data), these proportions of double and single signal sperm are highly significantly different from 1:1 (χ^2 ^= 14.96, P < 0.001). Thus they reveal that high Spam1 expression is a result of enrichment of transgene-bearing sperm or transgenic overexpression. The fact that a third of the sperm analyzed had a single signal can be explained by the overlapping peaks in the bimodal distribution (Fig. 2b), resulting in collection of some of the subpopulation without Spam1 overexpression.

We also analyzed the CDs in Tg8 hemizygotes for the presence of the closely related Hyal5 protein encoded by *Hyal5 *which is present on the transgene. In Fig. 3c-B and 3c-C we show large amounts of Hyal5 in CDs at the neck of sperm and its absence on the head, similarly to what was seen for Spam1. These CDs are distinctly different from normal CDs which were devoid of the protein (Fig. 3c-A), and were not seen in sperm from normal males. The overexpression of Hyal5 and its presence in retained CDs are consistent with the findings for Spam1 in Tg8 mice.

To test the LOS hypothesis using the *Spam1 *null carriers, caput sperm from sexually mature carriers and wild-type males were analyzed by flow cytometry for Spam1 quantities. There was a bimodal distribution including a subpopulation of sperm with only the baseline fluorescence, as can be seen for two animals in Fig. 2C, which was not seen for caput sperm in wild-type animals (data not shown). Note that in Fig. 2C the position of the two peaks in A and B, are consistent with the presence of sperm carrying the null allele (background or baseline fluorescence) and those with the normal allele.

**Figure 4:**
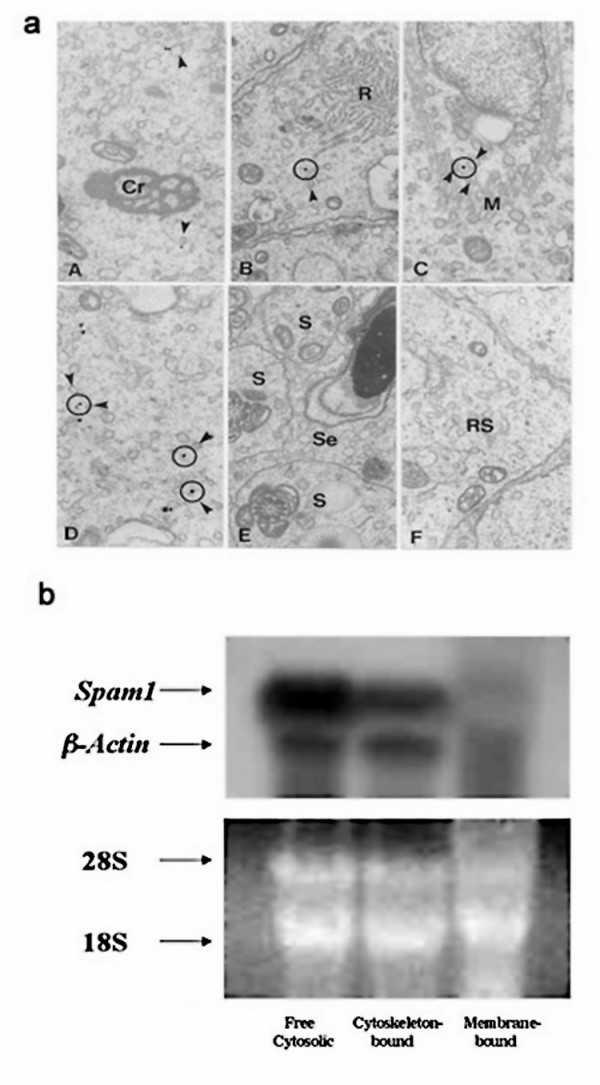
*Spam1 *transcripts which are compartmentalized are absent from the bridges and are associated with the cytoskeleton. **a) **EM autoradiography of seminiferous tubules after *in situ *hybridization with a tritiated (^3^H-labeled) *Spam-1 *antisense RNA probe. Note that silver grains are associated with the ER (arrowheads) but not with any other major spermatid structures such as the chromatoid bodies (A), the radial bodies (B) or the microtubules of the manchette (C). D is an intercellular bridge where the curvatures at the top and bottom represent the outer limits of the bridge. While some grains are seen in association with the ER in the vicinity, they are absent from the bridge. The circles centered over the silver grains include profiles of ER (arrowheads). (E) Late spermatids (S) and Sertoli cells (Se) are unreactive. (F) Shows the cytoplasm of round spermatid (RS) step 8 of a control section hybridized to a sense probe. Co, chromatoid body; Rb, radial body; M, manchette. X19,000 **b**. Northern hybridization of *Spam1 *and β-*actin *mRNAs in free cytosolic-, cytoskeletal-, and membrane-bound testicular RNA fractions. The fractions were separated by subcellular fractionation techniques. **A) **shows Northern blotting, while **B) **shows total RNA as a loading control with ethidium bromide staining. The presence of cytoskeletal-bound β-actin RNA in the free cytosolic fraction suggests that *Spam1 *in the latter could be present as a contaminant due to the preparation procedure.

We addressed the underlying mechanism for the TRD by performing ultrastructural studies to examine the precise localization of *Spam1 *transcript. Using *in situ *transcript hybridization with a ^3^H-labeled *Spam1 *antisense probe and electron microscopy to reveal the precise subcellular location of the RNA, we show that silver grains were compartmentalized. They were predominantly associated to the ER (Table 1). Conversely, the silver grains were not associated to structures such as the nucleus, chromatoid bodies or radial bodies (Table 1). Silver grains were sometimes located near the vicinity of intercellular bridges. However, they were associated to the ER, suggesting that the transcripts were not in transit but anchored to/near this organelle (Fig. 4a-D).

**Table 1:** Distribution of radioautographic silver grains in spermatids expressed as percentages (±SD)

	Steps 3–5	Steps 6–8	Steps 9–11
ER	85 ± 5	85 ± 2	87 ± 1
Assigned to ER	13 ± 3	14 ± 6	13 ± 1
Nucleus	0 ± 0	0 ± 0	0 ± 0
Chromatoid B^a^	0 ± 0	0 ± 0	0 ± 0
Radial Bodies	0 ± 0	0 ± 0	0 ± 0
Cytoplasmic B^b^	0 ± 0	0 ± 0	0 ± 0
Unknown origin	2 ± 0	1 ± 0	0 ± 0

^a ^Chromatoid bodies, ^b ^Cytoplasmic bridges. Some of the data in this table are taken from Molecular Reproduction & Development ^© ^copyright 2004 Wiley-Liss, Inc, A Wiley Company.

To further probe the subcellular location of the transcript and determine the nature of the anchoring, we performed biochemical fractionation of testicular RNA to identify cytoskeletal-associated, membrane-associated, and cytosolic-related fractions, as previously described [25]. These fractions were probed in Northern analysis with *Spam1 *cDNA, using β-actin (which is known to be cytoskeletal-bound) as an internal control. Fig. 4b shows that *Spam1 *mRNA is not membrane-associated, but was found sequestered with the cytoskeleton. The similar pattern for *β-actin *and *Spam1*, allows us to conclude that *Spam1 *is cytoskeletal-bound. Transcripts in the cytosol are either newly formed ones that have just exited the nucleus and have not yet been bound to the cytoskeleton, or those that are a contaminant of the preparation process.

**Figure 5:**
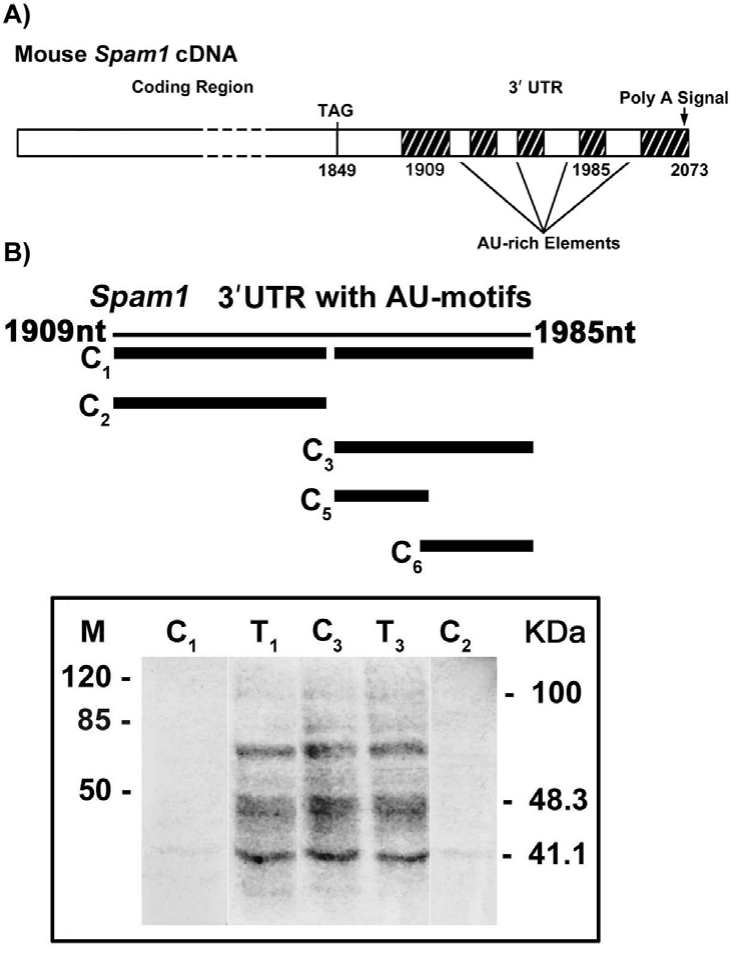
*Spam1 *RNA contains four AREs in the 3' UTR and UV-cross-linking reveals that one or two specifically bind testicular proteins. **A) **Four AU-rich motifs, 5' AUUUG...AUUUUA...AUUUUUA...AUUUUUG...3', are found in a 77 nucleotide sequence (nt 1909–1985) in *Spam1 *3' UTR. **B) **In the upper panel the thin line represents the 77 nt riboprobe sequence containing the AU motifs while the thick lines show the antisense DNA oligomers used in competition assays. C_1 _contains antisense oligos for all four AREs, C_2 _for the two at the 5' end of the sequence, C_3 _for the two at the 3' end, C_5_for the third from the 5' end, and C_6 _for the 3' end motif. The lower panel shows the results of *in vitro *label transfer by UV cross-linking and the RNA-protein complexes formed with the riboprobe and testicular protein extract, without competition, in Lanes T_1 _and T_3_. Lanes C_1 _and C_2 _show the disappearance of the complexes after competition with antisense oligos for all four motifs and the two at the 5' end, respectively. Lane C_3 _shows that the complexes are not diminished with competition with antisense oligos for the two 3' end AREs.

RNA-cytoskeletal binding has been shown to occur via AU-rich motifs in the 3' UTR [28]. Thus we searched *Spam1 *RNA sequence and identified four AREs in a 77 nucleotide (nt) sequence in the 3' UTR (nt 1909–1985), as shown in Fig. 5A. To investigate whether the sequence was a target for RNA-binding proteins that mediate cytoskeletal binding we generated a full-length 77 nt riboprobe for use in *in vitro *label transfer by UV-cross-linking. Testicular cytoplasmic proteins that bind specifically to the riboprobe were identified and are seen in Lanes T_1_, C_3 _and T_3 _in Fig. 5B. Addition of ~100-fold molar excess of unlabeled competitor antisense DNA oligomers for all four AREs (C_1_) virtually abolished RNA-protein complex formation as seen in Lane C_1 _(Fig. 5B), indicating the binding specificity of the ARE(s).

Similarly, pre-incubation of the riboprobe with unlabeled antisense DNA oligos for the two AREs in the 5' end of the probe (C_2,_) (prior to mixing with the protein extract) also abolished the binding as seen in Lane C_2 _(Fig. 5B), confirming the binding specificity. However, the addition of ~100-fold excess unlabeled antisense DNA oligos for the two AREs in the 3' end of the RNA (C_3_) did not diminish the formation of the RNA-protein complexes as seen in Lane C_3 _(Fig. 5B), indicating that these ARE's do not participate in the binding. When antisense oligos for these two 3' AREs were individually used in competition (C_5 _and C_6_), the formation of the RNA-protein complexes were also not diminished (data not shown). These results of *in vitro *binding are consistent with binding activity of *Spam1 *RNA, via one or both of the 5' AREs, to proteins that mediate cytoskeleton binding.

## Discussion

The results show a highly significant (P < 0.001) heritable TRD in Tg8 carriers, in favor of normal sperm and against transgene-bearing ones with 10 copies of *Spam1/Hyal5*. [The Mendelian 1:1 transmission ratio that was detected for carriers of the other transgenic lines can be attributed to the difficulty in obtaining overexpression of these genes due to naturally occurring antisense RNA [Zhang et al., submitted]. Importantly the caudal sperm population of Tg8 carriers showed a bimodal distribution, reflecting the presence of sperm with different quantities of Spam1. FISH analysis showed that the high Spam1-expressing sperm were enriched for the transgene, indicating transgenic overexpression of Spam1. That transgene-bearing sperm produced significantly less progeny than normal sperm shows that optimal levels of Spam1 and Hyal5 are required for fertility, similar to β1,4-galactosyl transferase where overexpression is associated with acrosome instability [29].

The failure of transgene-bearing sperm to effect fertilization in the expected ratio is a direct result of the retention of cytoplasmic droplets (CDs). A CD is an organelle with residual cytoplasm on the neck or tail of sperm. It results from a defect in the final stages of spermiogenesis and its presence in mature sperm renders them infertile [30,31]. It is interesting that sperm with overexpressed Spam1 and Hyal5 in CDs were lacking the proteins on the surface of the heads where they are normally found. Thus in addition to the sperm motility defects associated with CDs [32] there would be a decreased ability of penetration of the cumulus cells, leading to their infertility. It should be noted that in domestic animals CD-associated infertility has been shown to be due to poor passage through hyaluronate swim-up medium and failure to bind to the zona pellucida [33], both of which are functions of Spam1 [13]. However this is the first report of the presence of hyaluronidases in CDs, although a number of other enzymes have been identified in them [30].

The fact that the number of sperm with the enlarged CD phenotype is less than 50% and 100% in Tg8 hemizygotes and homozygotes, respectively, may be due to technical factors such as their loss during preparation [30]. It is also likely that the highly regulated *Spam1 *silencing which is mediated by antisense transcription [Zhang et al., submitted] could be responsible. This silencing would be adaptive since overexpression of Spam1 and Hyal5 leads to their mis-expression in CDs, which are abnormally retained and which lead to infertility. Thus in Tg8 carriers there are functionally different sperm within a male, due to their different quantities of Spam1 and Hyal5. It is not known if Spam1 and Hyal5 are overexpressed in the same CDs, consequently their co-localization will be investigated in future studies.

The finding of structurally and functionally different sperm in Tg8 transgene carriers is reminiscent of the findings for heterozygotes of spontaneous mutant alleles of *Spam1 *and is also consistent with compartmentalization of the RNA and protein [10]. The accumulation of the overexpressed Spam1 and Hyal5 protein in the CDs of trangene-bearing sperm is a result of a lack of transcript sharing between these sperm and those with the normal alleles. These observations on the transgenic model therefore provide support for the LOS Hypothesis in the etiology of TRDs.

Similarly, the LOS hypothesis is also supported by the findings from carriers of *Spam1 *null allele. Generated by insertion of a neo cassette in exon II (which contains the hyaluronidase domain) of *Spam1 *in the laboratory Tadashi Baba [17], null mice were shown to be fertile (despite a delay in cumulus penetration) due to the compensating effect of the redundant Hyal5 [17]. Carriers of *Spam1 *null showed a bimodal distribution of Spam1 in caput sperm, with one subpopulation having background levels of fluorescence, consistent with the presence of sperm with a null allele. Since Spam1 is expressed in the epididymis where it may be acquired by sperm during transit [18,19] caput, but not cauda, sperm would reflect the spermatid phenotype, and the finding from these sperm is supportive of a lack of transcript sharing.

To determine the mechanism for the lack of transcript sharing we focused on RNA compartmentalization. It has been proposed that mRNA localization facilitates protein sorting and that nascent polypeptide chain targeting of membrane proteins is a major mechanism that accounts for mRNA localization [34]. We show that 85% of the grains from *in situ *transcript hybridization localized to the ER and the remaining 15% could be assigned to this region. This indicates that the transcripts are not dispersed in the cytoplasm where they would gain ready access to the bridges. Table 1 shows that they are not associated with any major spermatid structure, such as the chromatoid bodies (which indicates that the RNA is not stored and is not translationally regulated), the radial bodies, or the microtubules of the manchette (Fig. 4a). Importantly, they are absent from the bridges although they may be in the vicinity. In this connection it should be pointed out that the absence of the transcript from the chromatoid bodies which have recently been seen to cross the bridges (Parvinen and Sassone-Corsi, personal communication), bolsters the evidence for the RNA compartmentalization.

The compartmentalization of *Spam1 *transcripts and their absence from the bridges suggest that they are anchored, and this would preclude sharing and support our LOS hypothesis. Based on the restricted location of the transcript at the ER, it was expected that Northern analysis would reveal its association with the membrane-bound fraction. However, this was not the case as *Spam1 *RNA was shown to be associated with the cytoskeletal fraction and therefore anchored. More importantly, we show that the transcript has AU-rich elements (AREs) in the 3' UTR that are known to bind cytoplasmic proteins (AUBPs) that mediate binding to the cytoskeleton. Interestingly, AREs are also present in the 3' UTR of the rodent-specific *Hyal5 *(Genbank Accession# ABO85680) which (at the nucleotide level) is 71% homologous to *Spam1 *with which it shares all functional domains [16]. Thus *Hyal5 *transcripts are likely to be cytoskeletal-bound and compartmentalized.

While cytoskeletal-binding may assist with anchoring and maintaining a pool of *Spam1 *and *Hyal5 *transcripts which can be recruited to the ER for co-translational assembly which occurs for membrane proteins [34], the data also suggest its involvement in posttranscriptional regulation. AREs are well-known to mediate RNA (in)stability by interacting with *trans*-acting proteins [35-37]. Further, their protein interaction which mediates cytoskeletal binding is known to be involved in mRNA turnover and posttranscriptional regulation of RNA [28,37]. We have recently observed that testicular *Spam1 *RNA may be stabilized by interactions with RNA-binding proteins [Zhang et al., submitted]. Therefore the presence of AREs in *Spam1 *RNA and the demonstration of their ability to specifically bind testicular proteins, potentially relates TRD with posttranscriptional regulation of the RNA. Simply put, the cytoarchitecture that facilitates the co-translational assembly of the transcript in the ER is involved in regulating mRNA decay and ultimately precludes RNA sharing via the bridges Taken together, our findings provide strong support for the LOS hypothesis. They also mechanistically relate RNA compartmentalization, mediated by cytoskeletal binding, and the regulation of the mRNA turnover to TRD.

TRD has been seen for a) the transmission of disease alleles such as delta F508 of the *CFTR *gene for cystic fibrosis [38] and b) the inheritance of the most common Robertsonian translocation [39], and is a phenomenon for which there is extensive evidence in the human genome [40]. Based on the diversity of genes involved, it is likely that there may be many different underlying mechanisms. However the findings in this study have uncovered, to our knowledge, the first molecular mechanism for a mammalian TRD.
